# Toward empirical correlations for estimating the specific heat capacity of nanofluids utilizing GRG, GP, GEP, and GMDH

**DOI:** 10.1038/s41598-023-47327-x

**Published:** 2023-11-25

**Authors:** Omid Deymi, Fahimeh Hadavimoghaddam, Saeid Atashrouz, Dragutin Nedeljkovic, Meftah Ali Abuswer, Abdolhossein Hemmati-Sarapardeh, Ahmad Mohaddespour

**Affiliations:** 1 Department of Mechanical Engineering, Shahid Bahonar University of Kerman, Kerman, Iran; 2 Institute of Unconventional Oil & Gas, Northeast Petroleum University, Daqing, 163318 Heilongjiang China; 3 Department of Chemical Engineering, Amirkabir University of Technology (Tehran Polytechnic), Tehran, Iran; 4 College of Engineering and Technology, American University of the Middle East, Egaila, 54200 Kuwait; 5 Department of Petroleum Engineering, Shahid Bahonar University of Kerman, Kerman, Iran; 6 Key Laboratory of Continental Shale Hydrocarbon Accumulation and Efficient Development, Ministry of Education, Northeast Petroleum University, Daqing, 163318 China; 7 Department of Chemical Engineering, McGill University, Montreal, QC H3A 0C5 Canada

**Keywords:** Engineering, Chemical engineering

## Abstract

When nanoparticles are dispersed and stabilized in a base-fluid, the resulting nanofluid undergoes considerable changes in its thermophysical properties, which can have a substantial influence on the performance of nanofluid-flow systems. With such necessity and importance, developing a set of mathematical correlations to identify these properties in various conditions can greatly eliminate costly and time-consuming experimental tests. Hence, the current study aims to develop innovative correlations for estimating the specific heat capacity of mono-nanofluids. The accurate estimation of this crucial property can result in the development of more efficient and effective thermal systems, such as heat exchangers, solar collectors, microchannel cooling systems, etc. In this regard, four powerful soft-computing techniques were considered, including Generalized Reduced Gradient (GRG), Genetic Programming (GP), Gene Expression Programming (GEP), and Group Method of Data Handling (GMDH). These techniques were implemented on 2084 experimental data-points, corresponding to ten different kinds of nanoparticles and six different kinds of base-fluids, collected from previous research sources. Eventually, four distinct correlations with high accuracy were provided, and their outputs were compared to three correlations that had previously been published by other researchers. These novel correlations are applicable to various oxide-based mono-nanofluids for a broad range of independent variable values. The superiority of newly developed correlations was proven through various statistical and graphical error analyses. The GMDH-based correlation revealed the best performance with an Average Absolute Percent Relative Error (AAPRE) of 2.4163% and a Coefficient of Determination (R^2^) of 0.9743. At last, a leverage statistical approach was employed to identify the GMDH technique’s application domain and outlier data, and also, a sensitivity analysis was carried out to clarify the degree of dependence between input and output variables.

## Introduction

Nanoparticles, tiny species with an average size of 1–100 nm, are classified in various aspects such as their shape (or morphology), size, and physicochemical characteristics. Aluminum (Al), silver (Ag), titanium (Ti), and other metals, as well as oxide-based or non-metallic materials like copper-oxide (CuO), titanium-dioxide (TiO_2_), aluminum-oxide (Al_2_O_3_), and so on, can all be found in the nanoparticles category. When nanoparticles are mixed in conventional base-fluids, they generate novel compounds known as nanofluids with distinct properties^1^. Water (W), ethylene glycol (EG), water-ethylene glycol mixture (W+EG), propylene glycol (PG), glycerol (GC), and engine oil (EO) are just a few examples of the wide variety of base-fluids that might be employed. Finally, nanofluids can be prepared in the forms of mono-nanofluids (e.g., Ag/W or TiO_2_/EG), binary hybrid nanofluids (e.g., SiO_2_–CuO/EG), or ternary hybrid nanofluids (e.g., Al_2_O_3_–CuO–TiO_2_/W), according to their expected purpose and application^2^.

The utilization of nanofluids for a variety of practical purposes is a popular and developing scientific field that is getting more attention day by day. Some of the most important applications of nanofluids, which can be observed in numerous research studies and publications, include the following:

- heat exchangers with different configurations (such as shell and tube, finned tube, double pipe, etc.)^3^,
- various types of sun-based systems (especially solar collectors and photovoltaic panels)^4,5^,
- cooling systems of vehicle engines and electronic components^6,7^,
- cooling of nuclear reactors^8^,
- drug-delivery and biomedical systems^9^,
- enhanced oil recovery (EOR) techniques^10^,
- common machinery processes (milling, drilling, grinding, and turning)^11^.

Until now, many analytical and numerical calculations have been performed on fluid-flow problems based on various kinds of nanofluids, mainly in research pertaining to the sectors of energy, electronics, and medicine. For instance, Jia et al.^12^ carried out the numerical analysis of a glazed photovoltaic-thermal (PV/T) collector employing two kinds of coolant nanofluid (Al_2_O_3_/W and TiO_2_/W) with the aim of investigating its thermal and electrical performances. McCash et al.^13^ mathematically conducted a comparative analysis for the peristaltic flow of two distinct nanofluids (Ag–Cu/W and Cu/W) inside an elliptical duct with sinusoidally advancing boundaries. Shahzad et al.^14^ analyzed the electro-osmotic flow of a blood-based hybrid nanofluid consisting of single- and multi-walled carbon nanotubes (SWCNT and MWCNT) through a multiple-stenosed artery with permeable walls. Alghamdi et al.^15^ mathematically modeled the two-dimensional magneto-hydrodynamics (MHD) flow based on the Cu–Al_2_O_3_/W hybrid nanofluid between two co-axial stretchable disks and numerically assessed it using the finite element method (FEM). Baig et al.^16^ presented exact analytical solutions for the stagnation-point flow of water-dispersed MWCNTs passing across a heated stretching cylinder.

Nanofluids have exceptional properties compared to normal fluids, which makes them a preferred choice in the field of mass and heat transfer phenomena. Following the stabilization of nanoparticles in the base-fluid after mixing together, the thermophysical properties of the resultant nanofluid, such as viscosity (*μ*), thermal conductivity (*k*), specific heat capacity (*C*_*p*_), and density (*ρ*), can be significantly altered. Several research efforts have been conducted on this topic in order to determine what other factors affect nanofluid properties and how these properties behave under different operational conditions. The present study only looks into changes in the specific heat capacity of mono-nanofluids (*C*_*p,nf*_) as a function of the specific heat capacity of nanoparticles (*C*_*p,np*_) and base-fluids (*C*_*p,bf*_), temperature (*T*), particle volume fraction (*ϕ*_*v*_), and average particle size (*d*_*np*_).

Recently, many researchers have been attempting to establish the specific heat values of nanofluids in various ways, such as by performing experimental tests, applying machine learning techniques (black-box models), and developing empirical mathematical relationships (white-box models). In 2008, Vajjha and Das^17^ released an empirical correlation for estimating the specific heat capacity of Al_2_O_3_/W+EG nanofluid as a function of temperature (313–363 K) and volume concentration (2–10 vol%). Their experimental measurements served as the basis for this cubic polynomial correlation. In addition, by comparing their measurements with estimated values achieved from the theoretical model of Pak and Cho^18^, they observed an acceptable level of agreement, with maximum and average deviations reported as 22% and 15%, respectively. In another investigation conducted in 2009, Vajjha and Das^19^ created a specific heat correlation using the experimental data related to SiO_2_/W, ZnO/W+EG, and Al_2_O_3_/W+EG nanofluids. Their correlation took into account four variables containing specific heat capacity of both nanoparticles and base-fluids, temperature, and particle volume concentration. This correlation set a defined range for each of the first two variables, and its constants for the three types of nanofluids were different. In a subsequent study by Vajjha and Das^20^ in 2012, they updated the correlation proposed in reference^19^ by incorporating the experimental data of CuO/W+EG nanofluid and making the temperature term dimensionless via entering a reference temperature. In 2013, Barbés et al.^21^ assessed the specific heat capacity of Al_2_O_3_/EG and Al_2_O_3_/W nanofluids as a function of temperature and volume fraction. The researchers also developed a simple linear regression model that suited their experimental data well. In the next year, they performed another similar research^22^ on CuO/W and CuO/EG nanofluids. In 2015, Cabaleiro et al.^23^ studied the specific heat changes of five distinct metal-oxide-based nanofluids at high concentrations. These nanofluids contained ZrO_2_, ZnO, and MgO in pure EG, as well as ZrO_2_ and ZnO in the W+EG mixture (50:50 vol%). Finally, the researchers developed a specific heat correlation as a function of the nanoparticle and base-fluid specific heat capacities and particle volume fraction. In another research, Sekhar and Sharma^24^, while studying the specific heat capacity of Al_2_O_3_/W nanofluid at low concentrations (0.01–1 vol%), proposed a specific heat correlation in accordance with 81 experimental data-points of water-based Al_2_O_3_, SiO_2_, TiO_2_, and CuO nanofluids collected from other researches. This regression equation was valid in the particular range of nanofluid temperatures, nanoparticle diameters, and volume fractions. It was found that the calculated values and experimental data were well compatible with each other, such that a deviation range between −8% and +10% was obtained. Satti et al.^25^, after evaluating the specific heat capacities of five various nanofluids consisting of CuO, ZnO, SiO_2_, TiO_2_, and Al_2_O_3_ nanoparticles suspended in a W+PG mixture (40:60 wt%), developed a correlation onto 610 measured data by employing the Minitab statistical software. Popa et al.^26^, according to their experimental measures on the specific heat capacity of CuO/W, Al_2_O_3_/W, and Al_2_O_3_/EG nanofluids, recalibrated the correlation developed by Vajjha and Das^19^ and captured its new coefficients based on the analyzed nanofluids. The authors reported excellent agreement with a maximum Average Relative Error of nearly 2%. Moldoveanu and Minea^27^, based on their examinations measuring the specific heat capacities of TiO_2_/W, SiO_2_/W, and Al_2_O_3_/W nanofluids, modified Sekhar and Sharma’s^24^ equation to propose another correlation. Its validity was limited to a range of room temperatures and volume fractions of less than 5%. The average deviation of the specific heat values estimated via their correlation was reported to be roughly 11% when compared to the experimental results. In 2020, Çolak et al.^28^ presented a correlation with an average error of –0.005% utilizing a database with 1287 data-points made up of volume fraction and temperature (as independent variables) and experimental specific heat values of Cu–Al_2_O_3_/W hybrid nanofluid. Gao et al.^29^ proposed a correlation that was fitted by the experimental specific heat capacities related to the hybrid nanofluid of GrapheneOxide–Al_2_O_3_/W with mass fractions of 0.05–0.15 wt% at a temperature range of 20–70 °C.

Since the specific heat capacity of nanofluids relies on various conditions and factors, it is often time-consuming, expensive, and difficult to quantify this property accurately via experimental procedures. To determine the specific heat capacity values of mono-nanofluids, robust machine-learning methods were employed in our recent study^30^, but the current work intends to provide novel empirical correlations. The literature review clearly indicates that the number of correlations developed so far by other researchers is limited. On the other hand, old correlations are only valid and suitable for certain nanofluids and only cover a restricted range of independent variables. As a result, there is still an opportunity for advancement in this subject, and it is conceivable to provide more comprehensive correlations with fewer constraints that can be applied to an extensive collection of nanofluids.

The current study’s primary aim is to provide novel and precise mathematical correlations for estimating the specific heat capacity of mono-nanofluids. It should be noted that the considered mono-nanofluids have been selected only based on the oxide-based or non-metallic nanoparticles dissolved in conventional base-fluids. For this purpose, an extensive database consisting of experimental data taken from the literature is utilized (the same one involved in our previous study^30^), and finally, four correlations are created using robust soft-computing procedures. To evaluate and compare the quality of the present correlations with prior ones suggested by others, various statistical and graphical error analyses are applied. The principal advantage of these new mathematical equations is that they have considerably mitigated the limitations of the earlier correlations. Therefore, they are applicable to many different oxide-based mono-nanofluids and a broad range of independent input variables.

### Dataset specifications

The specific heat capacity of mono-nanofluids (*C*_*P,nf*_) can be defined as a function of average particle size (*d*_*np*_), particle volume fraction (*ϕ*_*v*_), temperature (*T*), nanoparticle specific heat capacity (*C*_*P,np*_), and base-fluid specific heat capacity (*C*_*P,bf*_). Accordingly, 2084 data-points related to the experimental values of *C*_*P,nf*_ were collected from diverse sources (19 references) available in the literature^17,19,21–23,25,29,31–42^. Before generating the final flawless dataset, necessary corrective actions were carried out in the data pre-processing stage, such as data cleaning, data integration, and data reduction, to ensure the establishment of more accurate estimations. Table S1 (in the Supplementary File) lists the references used to extract the required data, the characteristics related to the various types of nanoparticles and base-fluids available in the final database, as well as the ranges of input (independent) and output (dependent) variables. Additionally, Table S2 (in the Supplementary File) provides the descriptive statistics for each of the variables included in the database. It should be mentioned that the information given in these two tables was also reported in our prior article^30^. Moreover, box-and-whisker plots and frequency distribution histograms related to the six variables available in the database were drawn in previous work^30^, which are avoided here.

### Description of techniques

The modeling and prediction of complex or unknown systems’ behavior employing input–output data is a common use of system identification techniques. Because of this, there is now much more interest in soft-computing techniques, which deal with data processing in uncertain and inexplicit environments. Many scientific studies have discussed the application of evolutionary algorithms as efficient soft-computing tools for system identification^43–46^.

Due to the data-driven nature of all soft-computing strategies, having more data leads to a more reliable and comprehensive model. In this regard, the present study covers four popular techniques for creating distinct mathematical models to estimate the specific heat capacities of mono-nanofluids. These techniques include Generalized Reduced Gradient (GRG), Genetic Programming (GP), Gene Expression Programming (GEP), and Group Method of Data Handling (GMDH), which are all covered with further details in the following subsections.

The specific algorithms adopted in this study were essentially chosen based on their suitability and ability to address the research goal, their compatibility with the quantity and quality of data-points contained within the dataset, and their own unique strengths and weaknesses. The successful usage and efficient performance of these techniques have been proven across a variety of investigations conducted in numerous fields of engineering sciences^47–54^.

### A: Generalized Reduced Gradient (GRG)

One of the typical strategies used to solve multi-variable problems is the generalized reduced gradient (GRG). In this technique, the decision variable is the vector of *X*(*x*_1_,*x*_2_,…,*x*_*n*_), and the constraints are the functions of *g*_1_,*g*_2_,…,*g*_*m*_. The objective function or any constraint function can be linear or non-linear. Additionally, it’s possible that the ranges can be undefined. If no constraints exist, the problem is called an unconstrained optimization problem. It should be noticed that the lower and upper limits of the variables do not act as additional constraints, but are applied separately to the given program^55^.

The GRG technique uses the first-order partial derivatives of each function *g*_*i*_ with respect to the variables *x*_*i*_, which are calculated by approximation of the forward or central finite difference. The program is executed according to the simulator’s initial values. If the values provided by the simulator do not satisfy all constraints *g*_*i*_, the first step of optimization starts, and in this situation, the objective function equals the sum of deviations from the constraints plus a fraction of the problem’s objective function. This optimization ends with a message that indicates the practicality or impracticality of solving the problem. It should be noted that in some cases, the impractical response is due to the limitation of the program in local minima, which can be corrected by changing the initial value and re-executing. In the next step, the complete optimization cycle is performed, and the final report is obtained^54,56^.

### B: Genetic Programming (GP)

**Figure 1:**
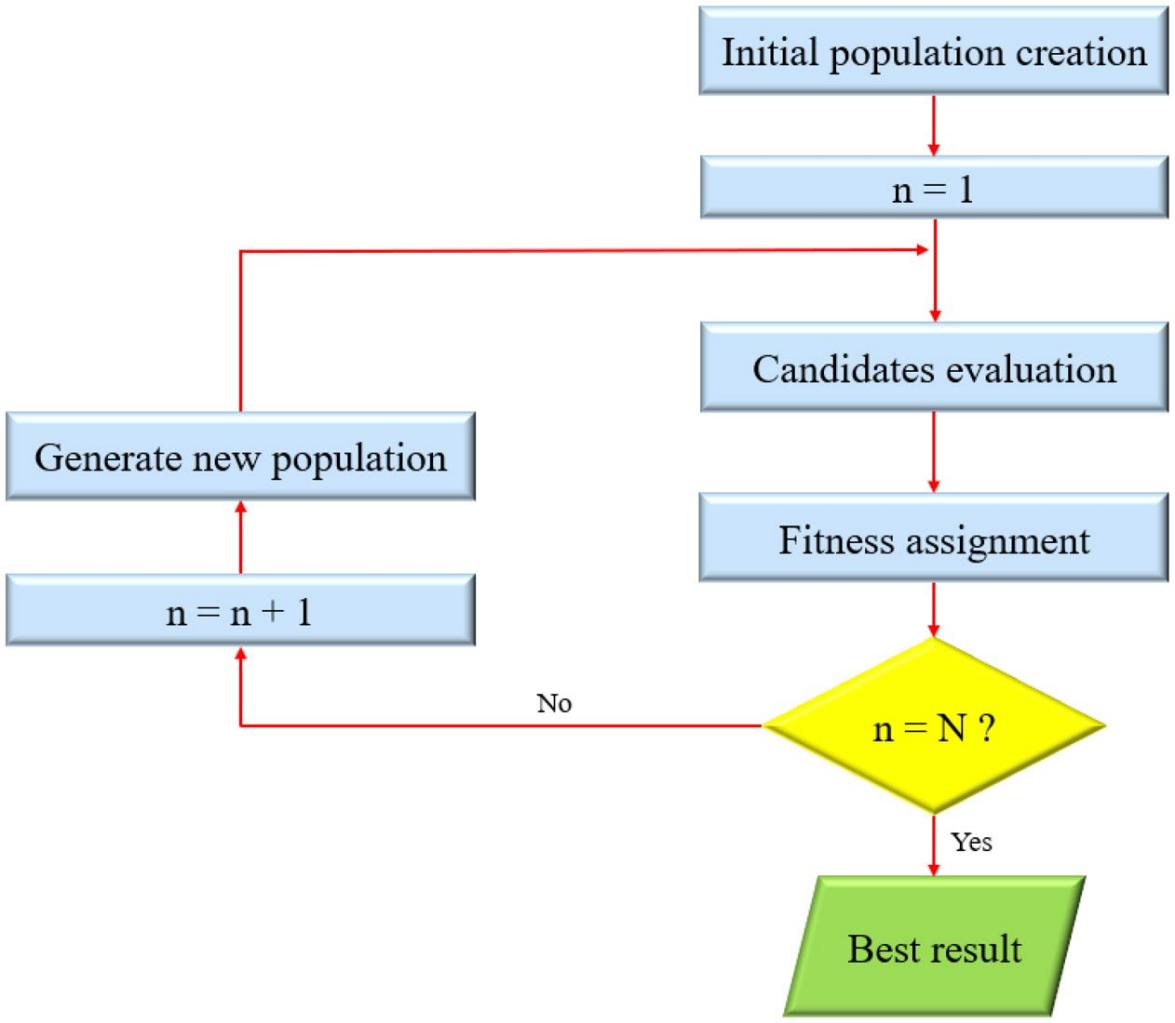
A flowchart of the GP technique.

A subset of the expanding family of evolutionary algorithms, namely genetic programming (GP), involves a population of randomly generated computer programs (Fig. 1). GP applies natural evolution-inspired search principles to a variety of diverse problems, particularly parameter optimization^57^. The concept of genetic programming clarifies a branch of artificial intelligence (AI) study that is concerned with the evolution of computer programming codes. The evolution process operates artificially in a way that is comparable to how living organisms evolve naturally, but most of its complicated details have been summarized^57^.

GP is an organized procedure for getting machines to solve a problem automatically, starting with a high-level statement that must be executed. This technique is considered a systematic and domain-independent algorithm that genetically reproduces a population of programs for problem-solving^58,59^. Besides, GP is an AI framework that imitates natural selection to identify the best prediction. This iterative procedure chooses the most suitable descendant to pass and regenerate at each new step of the algorithm^60^. GP has been successfully employed for a great number of various tasks, including data-mining^61^, pattern recognition^62^, and artificial neural network (ANN) synthesis^63^.

### C: Gene Expression Programming (GEP)

**Figure 2:**
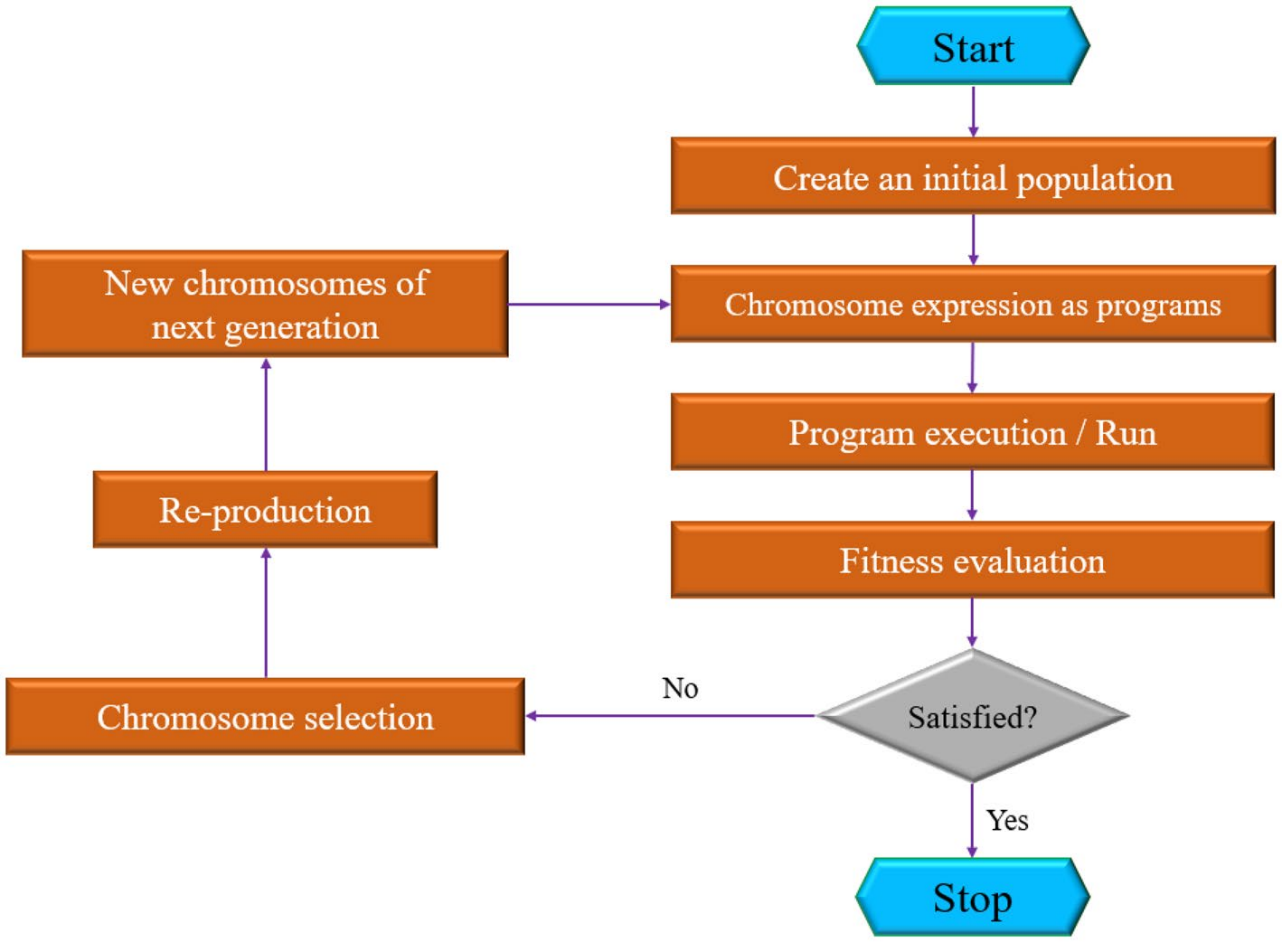
A flowchart of the GEP technique.

In 1999, *Ferreira* first presented the concept of Gene Expression Programming (GEP) as a strong soft-computing technique^64,65^. It is an advanced and upgraded version of GP and employs two major computational components in its structural computations for regression tasks, namely chromosomes (genotype) and expression trees (phenotype). However, the GP structure utilizes nonlinear forms of responses, namely parse trees. Actually, the chromosomes detect the selection or initial predictions for a particular problem, in which case using an interpretation strategy will allow the expression trees to provide more accurate solutions to the issues^66–68^. The simple flowchart related to this technique is shown in Fig. 2.

GEP, as a full-grown genotype/phenotype technique, develops computer programs encoded in fixed-length linear chromosomes. The structure of linear chromosomes makes possible the beneficial and unrestricted activity of crucial genetic operators like recombination, transposition, and mutation. Besides, GEP contributes a similar form of tree description and illustration to GP in order to retrace readily the stages accomplished by GP and discover effortlessly new boundaries established by exceeding the phenotype threshold^66^.

In recent decades, GEP, as a preferred and recognized evolutionary approach for automatically creating computer programs, has developed and advanced quickly, such that a variety of advanced GEPs have been suggested for real-world applications that are proliferating rapidly^69^.

### D: Group Method of Data Handling (GMDH)

**Figure 3:**
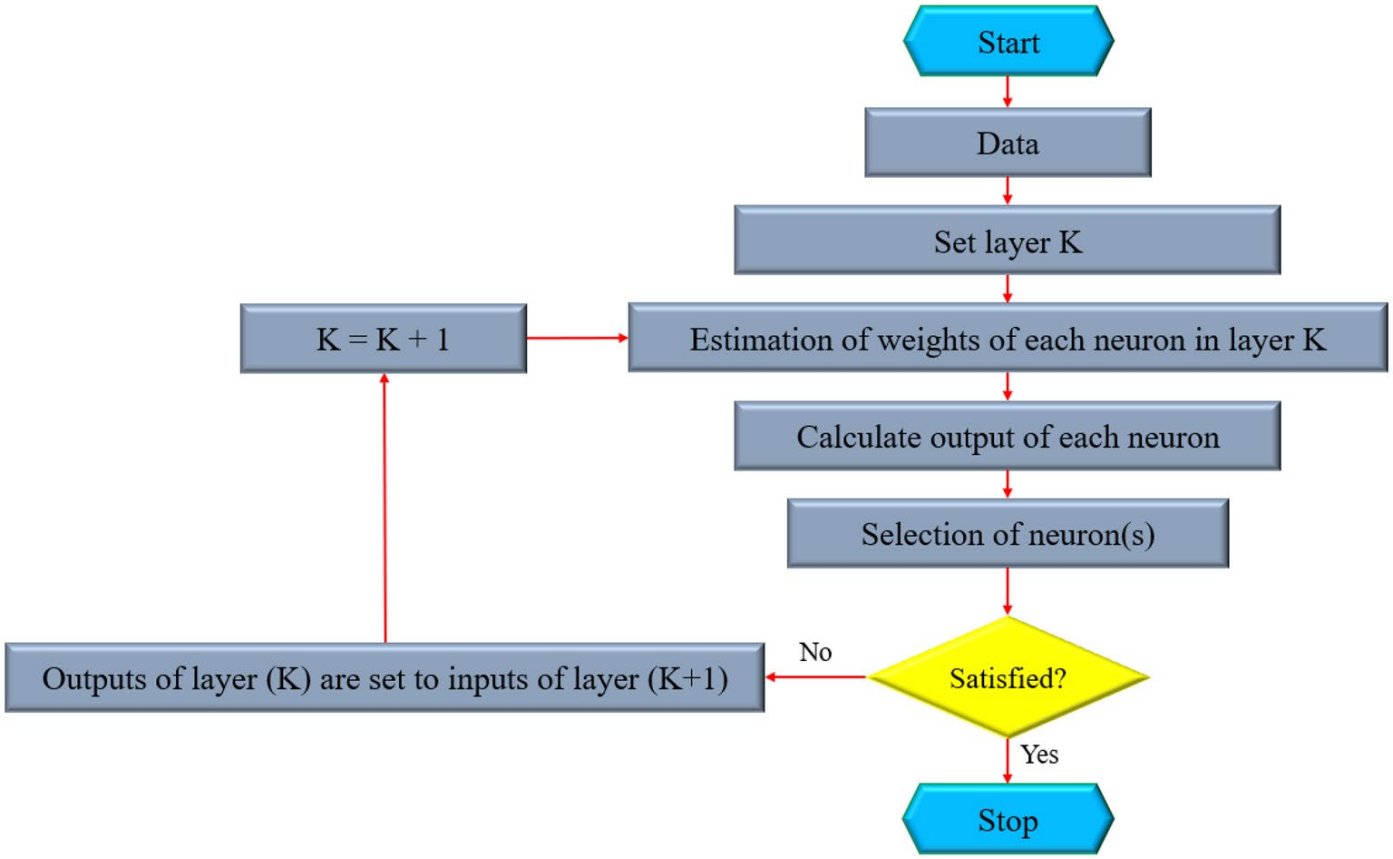
A flowchart of the GMDH technique.

A novel computational method for managing complex nonlinear computer tasks, known as the group method of data handling (GMDH), was first developed by *Ivakhnenko*. Figure 3 illustrates a functional flowchart for the GMDH technique. This technique can describe an explicit relationship between a mathematical model’s inputs and its corresponding output^70, 71^. This technique is one of the most satisfactory groups of artificial neural networks (ANNs) and is also known as a polynomial neural network (PNN). The inner layers of the GMDH technique contain a variety of independent nodes. This technique is provided in polynomial form, where all nodes in every layer are joined in couples through a quadratic polynomial and generate new polynomial-formed nodes in the subsequent layer, in accordance with the self-organizing principle^67,72^.

The GMDH technique is based on employing several nodes from the middle layers. Every node of GMDH returns a value by applying a quadratic polynomial approach that incorporates the preceding node^65,73^. As previously pointed out, the nodes of the next layers are produced using the quadratic polynomial functions that combine the nodes already present in the prior layers. The following formula represents the procedure of the GMDH technique^65,67,74^:

1 $$y_{i} = c + \mathop \sum \limits_{i = 1}^{w} \mathop \sum \limits_{j = 1}^{w} ... \mathop {\sum \, }\limits_{k = 1}^{w} c_{ij \ldots k} x_{i}^{n} x_{j}^{n} ...x_{k}^{n} \quad { ; } \quad \quad n = 1,2,..., 2^{m}$$

in which *x*_*ij…k*_ and *y*_*i*_ respectively depict input and output variables of the model, *m* and *w* respectively indicate the size of layers and the number of inputs, as well as *c* and *c*_*ij…k*_ stand for the polynomial factors.

By defining new nodal parameters (namely *P*_*i*_) for the situation of two nodes connected using a quadratic polynomial approach, the below formula is presented^65^:

2 $$P_{i}^{GMDH} = a_{0} + a_{1} x_{i} + a_{2} x_{j} + a_{3} x_{i} x_{j} + a_{4} x_{i}^{2} + a_{5} x_{j}^{2}$$

The least-squares method (LSM) is then used to minimize the variance between model predictions and actual data, as shown below^65^:

3 $$\delta_{j}^{2} = \mathop \sum \limits_{i = 1}^{{ \, N_{t} }} \left( { y_{i} - P_{i}^{GMDH} } \right)^{2 }\quad { ; }\quad \quad j = 1, 2, \ldots , \left( {\begin{array}{*{20}c} w \\ 2 \\ \end{array} } \right)$$

where *N*_*t*_ and *w* are the size of the training subset and the number of variables, respectively.

Later, the following general matrix will be created after obtaining the constants of Eq. (2) ^65^:

4 $$Y = A^{T} X \quad \quad {\text{or}} \quad \quad A^{T} = YX^{T} \left( {XX^{T} } \right)^{ - 1}$$

where *X* = [*x*_1_,*x*_2_,…,*x*_*w*_], *Y* = [*y*_1_,*y*_2_,…,*y*_*w*_], and *A* = [*a*_0_,*a*_1_,…,*a*_5_].

The model constants are attained over the training step. According to the below criterion, the testing subset is utilized to identify the optimum combination of two independent variables^70^:

5 $$\delta_{j}^{2} = \mathop \sum \limits_{{i = 1 + N_{t} }}^{ \, N} \left( {y_{i} - P_{i}^{GMDH} } \right)^{2 } { < }\varepsilon \quad { ; }\quad \quad j = 1, 2, \ldots , \left( {\begin{array}{*{20}c} w \\ 2 \\ \end{array} } \right)$$

## Mathematical models for calculation of nanofluids’ specific heat capacity

### Old correlations presented in the previous studies

Two primary theoretical models have been established to approximate the specific heat capacity values of nanofluids. The first one was developed by Pak and Cho^18^ in 1998 based on the ideal gases’ mixing theory according to Eq. (6). Another one was made by Xuan and Roetzle^75^ in 2000 on the basis of the thermal equilibrium of nanoparticles and base-fluid according to Eq. (7).

6 $$C_{P,nf} = \phi_{v} C_{P,np} + \left( {1 - \phi_{v} } \right)C_{P,bf}$$

7 $$C_{P,nf} = \frac{{\phi_{v} \rho_{np} C_{P,np} + \left( {1 - \phi_{v} } \right)\rho_{bf} C_{P,bf} }}{{\phi_{v} \rho_{np} + \left( {1 - \phi_{v} } \right)\rho_{bf} }}$$

Besides these two models, several other empirical correlations have been generated by various researchers in recent studies. Since the full description of these studies was detailed in the literature review part of the present work, only the developed correlations and their validity conditions are reported here:

- Vajjha and Das^17^:
  8 $$C_{P,nf} = A.\phi_{v} + B.\phi_{v} .T + C.\phi_{v} .T^{2} + D.\phi_{v} .T^{3}$$
  where *T* is in Kelvin and the coefficients *A*, *B*, *C*, and *D* are quartic polynomial functions of *ϕ*_*v*_. By applying Eq. (8), the specific heat capacity of Al_2_O_3_ nanoparticles dissolved in a 40:60 (wt%) water-ethylene glycol mixture can be calculated at volume concentrations of 2–10 vol% and a temperature range of 313–363 K.
- Vajjha and Das^19^:
  9 $$\frac{{C_{P,nf} }}{{C_{P,bf} }} = \frac{{(A.T) + B.\left( {\frac{{C_{P,np} }}{{C_{P,bf} }}} \right)}}{{C + \phi_{v} }}$$
- Vajjha and Das^20^:
  10 $$\frac{{C_{P,nf} }}{{C_{P,bf} }} = \frac{{A^{\prime}.\left( {\frac{T}{{T_{^\circ } }}} \right) + B.\left( {\frac{{C_{P,np} }}{{C_{P,bf} }}} \right)}}{{C + \phi_{v} }}$$

Equations (9) and (10) are applicable across temperatures of 315 < *T*(K) < 363 as well as volume fractions of 0 < *ϕ*_*v*_(%) ≤ 0.07 for nanofluids containing ZnO and 0 < *ϕ*_*v*_(%) ≤ 0.1 for nanofluids containing SiO_2_ and Al_2_O_3_, all dispersed in a mixture of water and ethylene glycol (40:60 wt%). Regarding the type of nanoparticles, Table 1 presents the constants of these two equations and the error values reported by the aforementioned authors.

**Table 1:** Characteristics of correlations (9) and (10) provided by Vajjha and Das^19,20^.

Nanoparticle	*A*	*A′*	*B*	*C*	Maximum error (%)	Average absolute error (%)
ZnO	0.0004604	0.12569	0.9855	0.2990	4.4	2.70
SiO_2_	0.0017690	0.48294	1.1937	0.8021	3.1	1.50
Al_2_O_3_	0.0008911	0.24327	0.5179	0.4250	5.0	2.28

- Sekhar and Sharma^24^:
  11 $$\frac{{C_{P,nf} }}{{C_{P,bf} }} = 0.8429\left( {1 + \frac{{T_{nf} }}{50}} \right)^{ - 0.3037} \left( {1 + \frac{{d_{np} }}{50}} \right)^{0.4167} \left( {1 + \frac{{\phi_{v} }}{100}} \right)^{2.272}$$
  Equation (11) is valid for water-based nanofluids comprising CuO, SiO_2_, TiO_2_, and Al_2_O_3_ and in the ranges of 20 < *T*_*nf*_ (°C) < 50, 15 < *d*_*np*_(nm) < 50, and 0.01 < *ϕ*_*v*_(%) < 4.00. The error range of this correlation was reported between –8% and +10%.
- Çolak et al.^28^:
  12 $$C_{P,nf} = \left( {A. \, T + B} \right)\left( {1 + C.\phi_{v}^{D} } \right)$$
  where *T* is temperature (°C) and *ϕ*_*v*_ is volume fraction (vol%). This correlation, which has been developed for Cu–Al_2_O_3_/W hybrid nanofluid, has constants and error values shown in Table 2.

**Table 2:** Characteristics of correlation (12) provided by Çolak et al.^28^.

*A*	*B*	*C*	*D*	Maximum error	Average error (%)
0.0002322	4.168	– 0.01175	0.6207	0.031	– 0.005
- Gao et al.^29^:
  13 $$C_{P,nf} = A + B.\phi_{m} + C.T + D.\phi_{m}^{2} + E.\phi_{m} .T + F.T^{2}$$

Equation (13) has been generated based on experimental data of GrapheneOxide–Al_2_O_3_/W hybrid nanofluids, in which *T* and *ϕ*_*m*_ are temperature (°C) and mass fraction (wt%), respectively. Table 3 lists the constants and the error value related to this equation.

**Table 3:** Characteristics of correlation (13) provided by Gao et al.^29^.

*A*	*B*	*C*	*D*	*E*	*F*	Maximum error (%)	R^2^
3.918	– 218.3	6.596 × 10^–3^	3.185 × 10^5^	5.278	– 5.35 × 10^–5^	0.86	0.983

### New correlations developed in the present study

#### (a) The GRG-based correlation:

Using the *solver* tool available in the *Data* tab of *Excel* software, various mathematical relationships with different numbers of constants can be created through the implementation of the GRG technique. For this purpose, a correlation in the form of Eq. (14) was developed with the aim of minimizing the average absolute percent relative error (AAPRE) regarding input and output variables (for the total dataset). According to the average and mode values related to the nanoparticle size and temperature variables, available in the descriptive statistics presented in Table S2 (in the Supplementary File), the reference values for this equation were considered to be *d*_o_ = 50 nm and *T*_o_ = 300 K.

14 $$C_{P,nf}^{{\text{GRG}}} = C_{P,bf} .\left[ {a_{0} + a_{1 \, } .\left( {\frac{{d_{np} }}{{d_{^\circ } }}} \right)^{{a_{2} }} + a_{3 \, } .\left( {\frac{{\phi_{v} }}{100}} \right)^{{a_{4} }} + a_{5} .\left( {\frac{T}{{T_{^\circ } }}} \right)^{{a_{6} }} + a_{7 \, } .\left( {\frac{{C_{P,np} }}{{C_{P,bf} }}} \right)^{{a_{8} }} } \right]$$

where *a*_0_ = – 1.459532, *a*_1_ = 1.191867, *a*_2_ = – 0.044737, *a*_3_ = – 1.889018, *a*_4_ = 1.014473, *a*_5_ = 1.172350, *a*_6_ = 0.102400, *a*_7_ = 0.202981, *a*_8_ = 0.960041

The correlations obtained using meta-heuristic techniques are as follows.

#### (b) The GP-based correlation:

15 $$C_{P,nf}^{{\text{GP}}} = b_{0} + b_{1} .\left\{ {b_{2} {.}\phi_{v} - \left[ {b_{3} + \exp \left( {b_{4} {.}C_{P,bf} } \right)} \right].\log \left[ \begin{gathered} b_{5} + b_{6} {.}T + \left( {b_{7} .\frac{T}{{d_{np} }} + b_{8} .\frac{1}{{\phi_{v} }}} \right).C_{P,np} \hfill \\ - \exp \left( {b_{9} {.}C_{P,bf} } \right) \hfill \\ \end{gathered} \right]} \right\}$$

where *b*_0_ = – 2.124005, *b*_1_ = – 0.071654, *b*_2_ = 0.786437, *b*_3_ = 8.280478, *b*_4_ = 0.474517, *b*_5_ = 9.110290, *b*_6_ = 0.814494, *b*_7_ = 1.277965, *b*_8_ = 0.989456, *b*_9_ = 0.772331

#### (c) The GEP-based correlation:

16 $$C_{P,nf}^{{\text{GEP}}} = c_{0} - \left[ {\left( {c_{1} {.}\phi_{v} + c_{2} {.}C_{P,np} } \right) - (c_{3} + c_{4} {.}C_{P,np} ).\left( {c_{5} - c_{6} {.}\frac{{d_{np} {.}\phi_{v} }}{T}{.}C_{P,bf} } \right)} \right]{.}C_{P,bf} - \left( {c_{7} - c_{8} {.}T} \right){.}C_{P,bf}^{ \, 2}$$

where *c*_0_ = 0.327155, *c*_1_ = 2.615432 × 10^–3^, *c*_2_ = 2.191448 × 10^–3^, *c*_3_ = 0.0559709, *c*_4_ = 5.341911 × 10^–3^, *c*_5_ = 12.798509, *c*_6_ = 0.555788, *c*_7_ = 0.0123802, *c*_8_ = 1.3806004 × 10^–4^

#### (d) The GMDH-based correlation:

17 $$C_{P,nf}^{{\text{GMDH}}} = 0.045762 + 0.798159 \, d_{0} + 0.170415 \, d_{1} + 1.046540 \, d_{0} \, d_{1} - 0.614356 \, d_{0}^{ \, 2} - 0.426339 \, d_{1}^{ \, 2}$$

where

17a $$d_{0} = - 8.53143 + 5.2795 \, d_{2} - 0.804102 \, d_{2}^{ \, 2} + 0.0791396 \, d_{2} \, C_{P,bf} + 0.638003 \, C_{P,bf}$$

17b $$d_{1} = - 0.645007 - 0.83654 \, d_{3} - 0.601428 \, d_{3}^{ \, 2} + 1.64147 \, d_{3} \, C_{P,bf} + 2.13504 \, C_{P,bf} - 1.07153 \, C_{P,bf}^{ \, 2}$$

17c $$d_{2} = 4.02018 - 0.0248309 \, d_{np} - 2.45377 \times 10^{ - 3} \, d_{np} \, \phi_{v} + 2.32343 \times 10^{ - 4} \, d_{np}^{ \, 2} + 6.5419 \times 10^{ - 3} \, \phi_{v}^{ \, 2}$$

17d $$d_{3} = 1.81744 + 1.19136 \, d_{4} - 1.44623 \, d_{5} - 0.0777568 \, d_{4} \, d_{5} + 0.290077 \, d_{5}^{ \, 2}$$

17e $$d_{4} = 2.208 - 0.0779644 \, \phi_{v} - 0.372357 \, C_{P,bf} + 2.96343 \times 10^{ - 3} \, \phi_{v} \, C_{P,bf} + 1.60252 \times 10^{ - 3} \, \phi_{v}^{ \, 2} + 0.195263 \, C_{P,bf}^{ \, 2}$$

17f $$d_{5} = - 8.27289 + 0.0301767 \, d_{np} + 0.0604513 \, T - 2.14838 \times 10^{ - 4} \, d_{np} \, T + 2.83985 \times 10^{ - 4} \, d_{np}^{ \, 2} - 6.68282 \times 10^{ - 5} \, T^{2}$$

## Results and discussion

### Evaluation and comparison of the correlations

In the current research, four new correlations—Eqs. (14) to (17)—were attained with the aim of estimating the specific heat capacity of mono-nanofluids by executing the GRG, GP, GEP, and GMDH techniques. On the other hand, three previously proposed Eqs. (6), (11), and (12) were selected and implemented on the entire employed database. Then, in order to compare the estimated outcomes of all seven equations with their corresponding experimental values and to evaluate the performance and accuracy of those equations, the following statistical parameters for train and test subsets (including 1667 and 417 data-points, respectively) and the total database (with 2084 data-points) were computed and declared according to Tables 4 and 5.

**Table 4:** The statistical parameters of old correlations presented in the previous studies.

Equation	APRE (%)	AAPRE (%)	RMSE	SD	R^2^
Equation (6): Pak and Cho^18^	– 8.1331	8.2695	0.3009	0.1048	0.9183
Equation (11): Sekhar and Sharma^24^	– 8.1348	16.1876	0.6092	0.2109	0.3045
Equation (12): Çolak et al.^28^	– 33.0155	33.1611	1.0837	0.4250	0.0692

**Table 5:** The statistical parameters of new correlations developed in the current study.

Technique	Dataset	APRE (%)	AAPRE (%)	RMSE	SD	R^2^
GRG [Eq. (14)]	Train	– 0.0893	2.9009	0.1346	0.0417	0.9549
	Test	– 0.2619	3.1357	0.1436	0.0455	0.9513
	Total	– 0.1239	2.9479	0.1365	0.0425	0.9542
GP [Eq. (15)]	Train	– 1.0456	3.0771	0.1314	0.0428	0.9588
	Test	– 0.8034	2.7447	0.1180	0.0376	0.9676
	Total	– 0.9971	3.0106	0.1288	0.0418	0.9607
GEP [Eq. (16)]	Train	– 0.5012	2.4082	0.1102	0.0362	0.9702
	Test	– 0.2091	2.1603	0.0935	0.0295	0.9790
	Total	– 0.4428	2.3586	0.1071	0.0349	0.9720
GMDH [Eq. (17)]	Train	– 0.1050	2.3974	0.1007	0.0334	0.9750
	Test	– 0.1351	2.4915	0.1065	0.0351	0.9716
	Total	– 0.1110	2.4163	0.1019	0.0338	0.9743

1. *Average Percent Relative Error (APRE)* This parameter determines the relative deviation of estimated data-points from the associated experimental values and is computed as:
  18 $$APRE = \frac{1}{n}\sum\limits_{i = 1}^{n} {\left[ {\frac{{\left( {C_{P,nf,i} } \right)_{exp.} - \left( {C_{P,nf,i} } \right)_{est.} }}{{\left( {C_{P,nf,i} } \right)_{exp.} }} \times 100} \right]}$$
2. *Average Absolute Percent Relative Error (AAPRE)* This is calculated similarly to APRE with the exception that the errors’ absolute values are considered when determining the final values of errors:
  19 $$AAPRE = \frac{1}{n}\sum\limits_{i = 1}^{n} {\left| {\frac{{\left( {C_{P,nf,i} } \right)_{exp.} - \left( {C_{P,nf,i} } \right)_{est.} }}{{\left( {C_{P,nf,i} } \right)_{exp.} }} \times 100} \right|}$$
3. *Root Mean Square Error (RMSE)* This parameter indicates the level of data scattering around the zero point and is computed as:
  20 $$RMSE = \sqrt {\frac{1}{n}\sum\limits_{i = 1}^{n} {\left[ {\left( {C_{P,nf,i} } \right)_{exp.} - \left( {C_{P,nf,i} } \right)_{est.} } \right]^{2} } }$$
4. *Standard Deviation (SD)* This parameter represents the amount of data scattering from the average value and is calculated as:
  21 $$SD = \sqrt {\frac{1}{n - 1}\sum\limits_{i = 1}^{n} {\left[ {\frac{{\left( {C_{P,nf,i} } \right)_{exp.} - \left( {C_{P,nf,i} } \right)_{est.} }}{{\left( {C_{P,nf,i} } \right)_{exp.} }}} \right]^{2} } }$$
5. *Coefficient of Determination* (*R*^2^) This parameter evaluates how well the experimental and estimated values match, as follows:
  22 $$R^{2} = 1 - \frac{{\sum\limits_{i = 1}^{n} {\left[ {\left( {C_{P,nf,i} } \right)_{exp.} - \left( {C_{P,nf,i} } \right)_{est.} } \right]^{2} } }}{{\sum\limits_{i = 1}^{n} {\left[ {\left( {C_{P,nf,i} } \right)_{exp.} - C_{P,nf,ave} } \right]^{2} } }} \quad { , }\quad \quad where\quad \, C_{P,nf,ave} = \frac{1}{n}\sum\limits_{i = 1}^{n} {\left( {C_{P,nf,i} } \right)_{exp.} }$$

As seen in Table 4, Eq. (6) can provide relatively good estimations of the specific heat capacity for mono-nanofluids available in the database. Such a consequence has also been mentioned in the research of Vajjha and Das^17,20^ and Murshed^31^, but it contradicts the findings of Zhou and Ni^76^ and Zhou et al.^77^. However, Eq. (11) has not shown a good performance due to the parameter-related limitations implied in Ref.^24^ and the broad range of the variables existing in the current work. In addition, a poor and unacceptable performance is observed from Eq. (12) because it was obtained based on Cu–Al_2_O_3_/W hybrid nanofluids, while the database used in the present study only comprises mono-nanofluids. This result is owing to the dissimilarity of stability levels and thermophysical properties found in mono and hybrid nanofluids caused by their structure-related differences.

**Figure 4:**
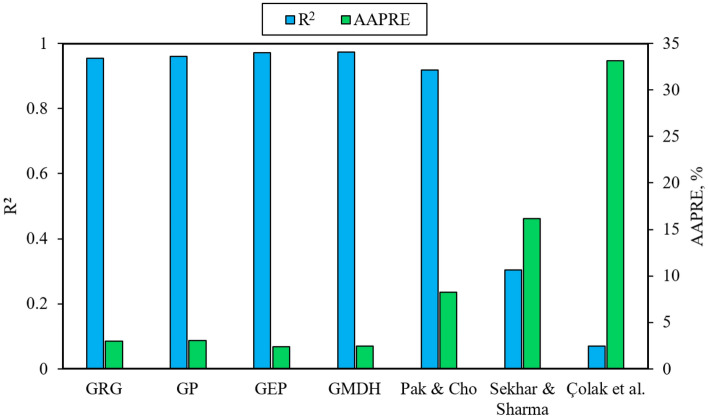
Graphical comparison of AAPRE and R^2^ values for the introduced correlations.

If the results of four novel correlations developed in the current study are compared with the estimations of the other three equations, it can be understood that these four correlations have very good accuracy in estimating the specific heat capacity of mono-nanofluids and have achieved acceptable error rates (AAPRE < 3.2% and R^2^ > 0.95). This issue is also depicted in Fig. 4 as a graphical analysis of the AAPRE and R^2^ parameters. When carefully observed, the best performance is found for correlations obtained using the GMDH and GEP techniques.

**Figure 5:**
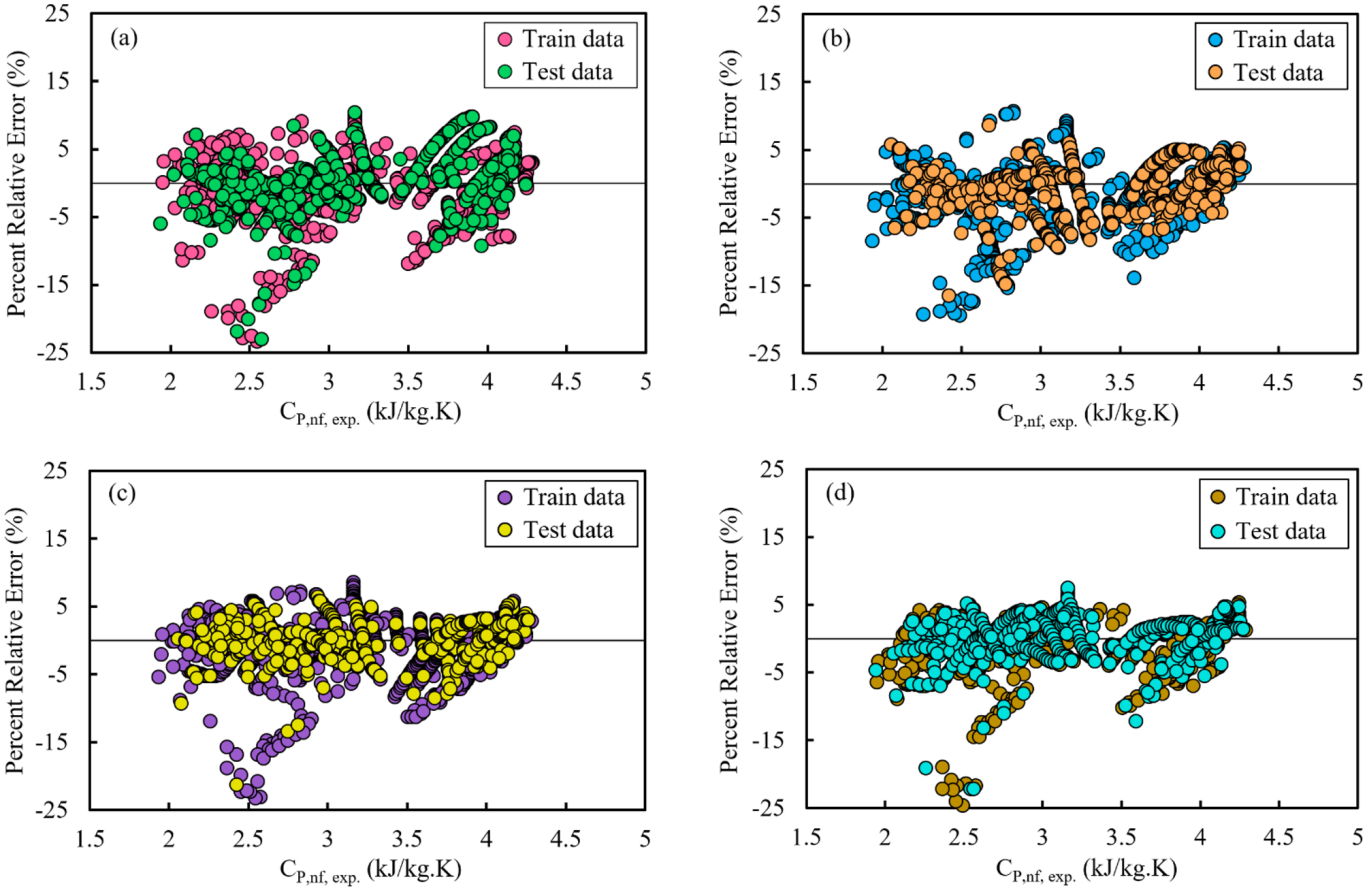
Percent relative error distribution against experimental values of C_P,nf_ for different correlations: (**a**) GRG, (**b**) GP, (**c**) GEP, and (**d**) GMDH.

Applying graphical error analysis is a suitable and useful tool for checking the performance of predictive models, especially when multiple various models are to be compared side by side. Figure 5 displays percent relative errors related to the estimated values of *C*_*p,nf*_ obtained from the GRG, GP, GEP, and GMDH techniques for the train and test subsets versus the experimental values of *C*_*p,nf*_. In this graph, the data-points are scattered along a line with zero error to shed light on the error pattern of estimation techniques. A large accumulation of data-points around this line indicates that a technique is highly accurate.

As illustrated in Fig. 5, a large percentage of the experimental data-points are overestimated by all techniques. In other words, a majority of the estimated values are greater than the experimental values, and therefore, the relative errors are negative numbers [see Eq. (18)]. However, Fig. 5d demonstrates that the GMDH technique overestimates a relatively small fraction of data-points (low to medium values of *C*_*p,nf*_) and performs a credible task in evenly estimating the other data-points. Although this technique would be suitable for estimating just medium- and large-range values of *C*_*p,nf*_, it still has almost an error tendency and needs to be utilized carefully.

According to Fig. 5d, the relative errors related to the *C*_*P,nf*_ values estimated by the GMDH technique are located near the line with zero error (within the error range of −24.6% to +7.5%). This technique has made more accurate and reliable estimates than those of the other techniques.

**Figure 6:**
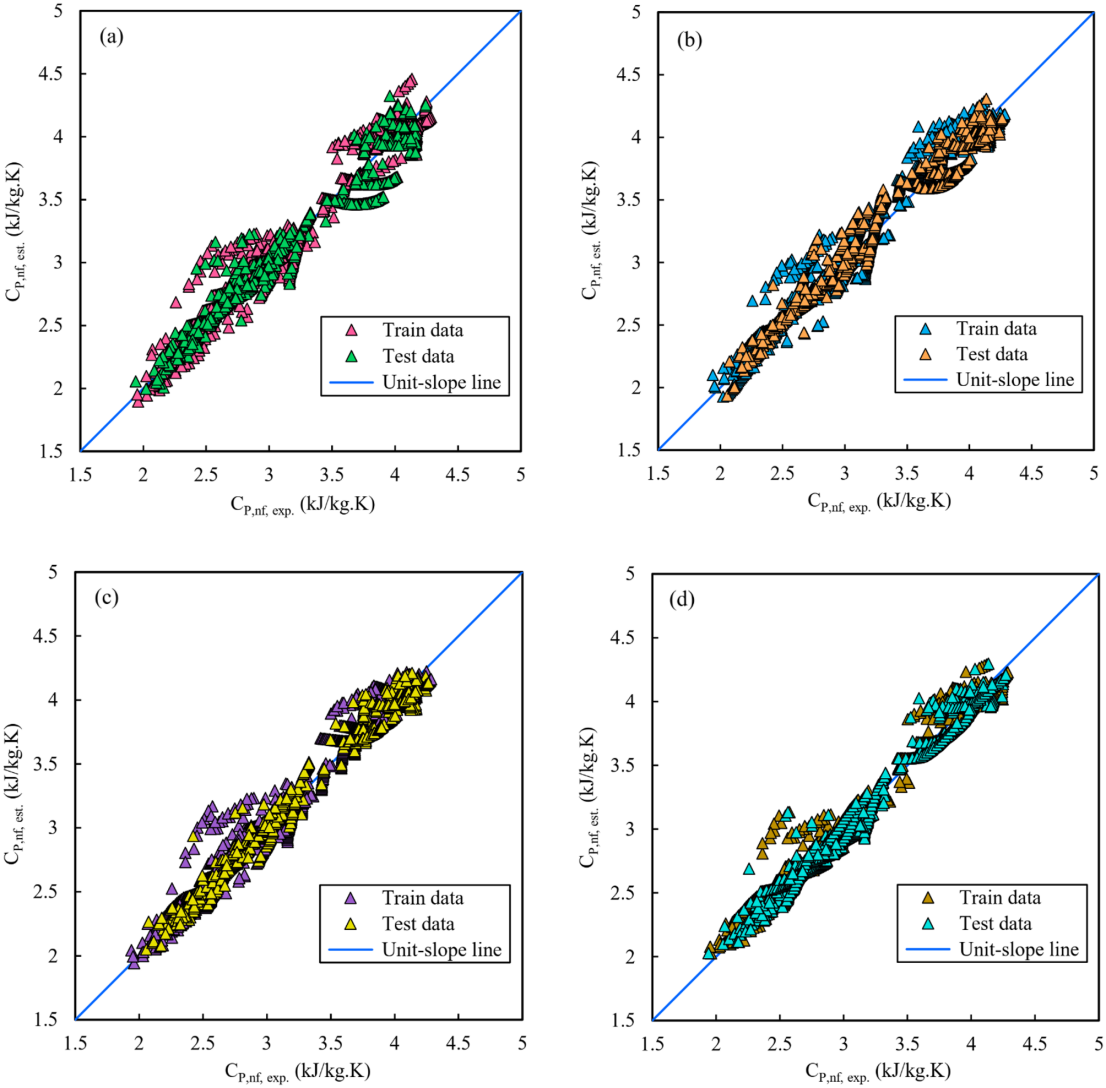
Cross-plots of the correlations developed for C_P,nf_ estimation: (**a**) GRG, (**b**) GP, (**c**) GEP, and (**d**) GMDH.

In order to undertake a more thorough analysis regarding the accuracy and precision of the suggested correlations, another visual evaluation was performed by utilizing a particular kind of scatter chart known as the cross-plot. In this method, the estimated data-points from a correlation are put against the experimental data and across a 45° line (known as a unit-slope line) passing the plot’s origin. Hence, the effectiveness of each correlation can be judged by how close the trend is to the 45° line. The cross-plots related to the developed correlations for *C*_*P,nf*_ estimation applying the GRG, GP, GEP, and GMDH techniques are presented in Fig. 6.

According to Fig. 6, a fairly packed accumulation of data-points along the unit-slope line is observed, particularly for the GMDH technique, whose outputs are in respectable agreement with the experimental data. Furthermore, the GRG technique has higher data deviations from the unit-slope line than the other techniques.

**Figure 7:**
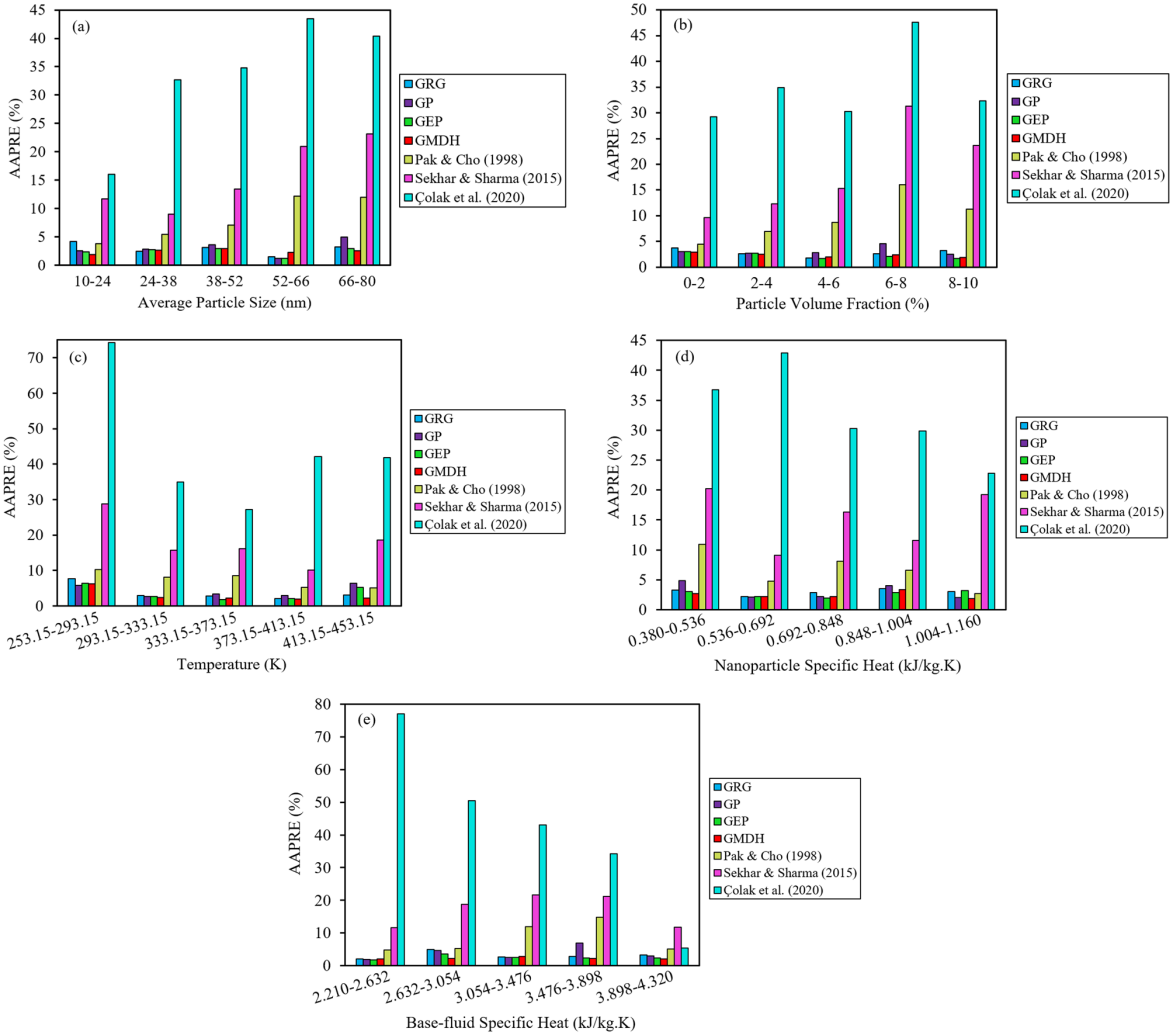
AAPRE distribution in different ranges of input variables for the suggested correlations.

In Fig. 7, the group-error distribution diagrams in terms of five input variables are exhibited for three old and four novel correlations. This method first involves classifying the input or independent variables into a certain number of ranges (or categories) according to the scope of their changes, after which the estimation error values for the target variable are determined and plotted in each range.

As depicted by Fig. 7a regarding the “average particle size” parameter, the lowest AAPRE values correspond to the range of 52–66 nm for the GRG, GP, and GEP techniques and the range of 10–24 nm for the GMDH technique. As shown in Fig. 7b regarding the “particle volume fraction” parameter, the lowest AAPRE values are achieved in the range of 4–6 vol% for the GRG and GEP techniques and the range of 8–10 vol% for the GP, GEP, and GMDH techniques. As can be seen in Fig. 7c concerning the “temperature” parameter, the lowest AAPRE values are attained in the range of 373.15–413.15 K for the GRG and GMDH techniques, the range of 293.15–333.15 K for the GP technique, and the range of 333.15–373.15 K for the GEP technique. As indicated in Fig. 7d regarding the “nanoparticle specific heat” parameter, the lowest AAPRE values are seen in the range of 0.536–0.692 kJ/kg.K (mainly Si_3_N_4_, TiN, CuO, and TiO_2_) for the GRG technique, the range of 0.692–0.848 kJ/kg.K (mainly Al_2_O_3_ and SiO_2_) for the GEP technique, and the range of 1.004–1.160 kJ/kg.K (mainly MgO) for the GP and GMDH techniques. As can be observed in Fig. 7e concerning the “base-fluid specific heat” parameter, the lowest AAPRE values are found for all four techniques of the GRG, GP, GEP, and GMDH in the range of 2.210–2.632 kJ/kg.K (mainly EthyleneGlycol). It is worth noting that with a growth in the heat capacity values of the base-fluids, the AAPRE values computed for the correlation belonging to Çolak et al.^28^ [i.e., Eq. (12)] have decreased.

**Figure 8:**
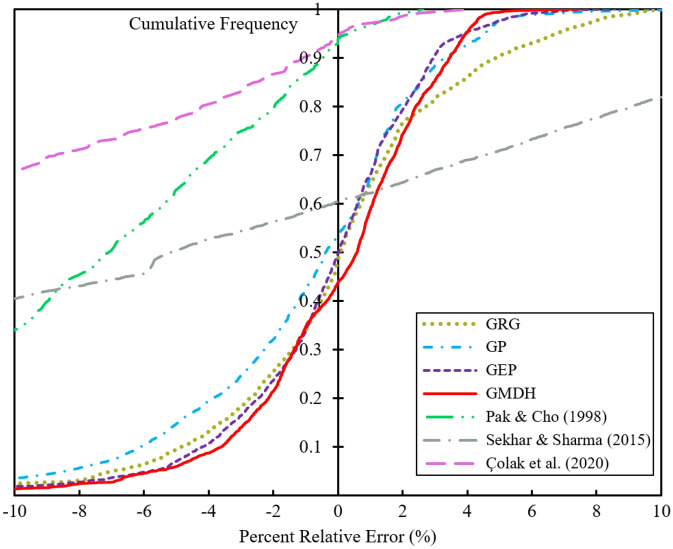
Cumulative frequency against percent relative error for suggested correlations.

Figure 8 illustrates a graphical analysis of cumulative frequency for evaluating the performance of the different correlations mentioned in the present study. According to this plot, the cumulative frequency of all data-points is depicted versus the percent relative errors to measure the number of data-points that the correlations can reliably estimate. From Fig. 8, it can be inferred that the GMDH technique surpasses the other techniques by successfully and effectively estimating more than 90% of total data-points with a relative error of −10% to +5%. Furthermore, the benefit and priority of the new correlations developed in this study are clearly visible compared to the other correlations.

### Outliers detection and applicability domain of a technique

A popular and effective method known as the “leverage statistical approach” is applied to find probable outliers (or unexpectedly aberrant data) and designate the applicability domain of the developed correlations^78–80^. For this purpose, this method generates a graph known as the “Williams plot” by determining two parameters, the Hat Matrix (*H*) and the Standardized Residuals (*SR*), utilizing the Eqs. (23) and (24) ^80–82^:

23 $$H = X\left( {X^{T} X} \right)^{ - 1} X^{T}$$

where the letter *T* indicates the transpose operator. *X* is a *N*_*t*_ × *N*_*i*_ matrix, where *N*_*t*_ and *N*_*i*_ depict the number of all data-points and technique inputs, respectively.

24 $$SR_{i} = \frac{{e_{i} }}{{RMSE\sqrt {1 - H_{ii} } }}$$

where *RMSE* refers to the root mean square error of the used technique, *e*_*i*_ denotes the discrepancy of the *i*-th estimated data-point from its associated experimental value, and the Hat Indices *H*_*ii*_ represent the diagonal components of the *H* matrix (i.e., *H*_*ii*_ = diag(*H*)).

Williams plot visually displays three important zones associated with the existence of valid data, out-of-leverage data, and suspected data (outliers) by revealing the relation between Standardized Residuals and Hat Indices. Valid data-points can be found in the ranges −3 ≤ *SR* ≤ +3 and 0 ≤ *H* ≤ H*. The leverage values equal to −3 and +3 for the parameter *SR* are called the cut-off metrics. The parameter H*, also referred to as the critical leverage or warning leverage, has the value 3(*N*_*i*_ + 1)/*N*_*t*_. The data-points that belong to the ranges −3 ≤ *SR* ≤ +3 and *H* > H* are recognized as “good high leverage” points, which are outside the applicability domain of the technique by which a correlation has been generated. The rest of the data-points with *SR* < −3 or *SR* > +3 (independent of *H* values) are classified as suspected data or outliers. Due to their negative effect on the slope and intercept of the constructed regression line, they are considered a risk to reliable modeling and are also called “bad high leverage” points^78–83^.

**Figure 9:**
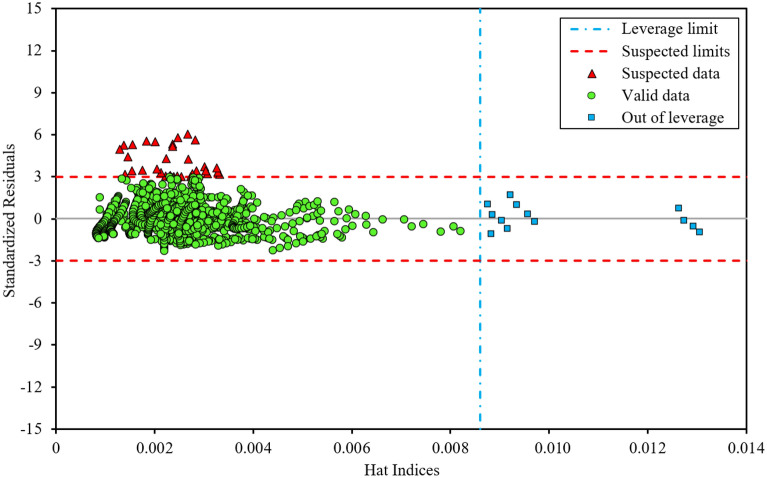
Williams plot related to the correlation developed by the GMDH technique.

Figure 9 exhibits the Williams plot for the best-proposed technique (i.e., GMDH) based on the total dataset employed in the current study. This graph shows that the GMDH technique detects 1.68% of the 2084 data-points (35 red triangular marks) as probable outliers. Furthermore, just 0.62% of the 2084 data-points (13 blue square marks) are found to be outside the applicability domain of the GMDH technique. As a result, due to the existence of a substantial portion of valid data-points (green circular marks) in the ranges −3 ≤ *SR* ≤ +3 and 0 ≤ *H* ≤ 0.0086, the accuracy and reliability of this technique are confirmed again.

### Sensitivity analysis

Performing sensitivity analysis can include several purposes, such as detecting errors existing in the model’s structure, determining the grade of relation between input and output variables of a model, and refining a model by discarding inputs that have little or no effect on its output. The relevancy factor (*r*) is a parameter that is used to assess how much every input variable influences the output of a model. This factor’s value can vary from −1 to +1, and the greater its absolute value for an input variable, the higher its impact on the model’s output^30^.

**Figure 10:**
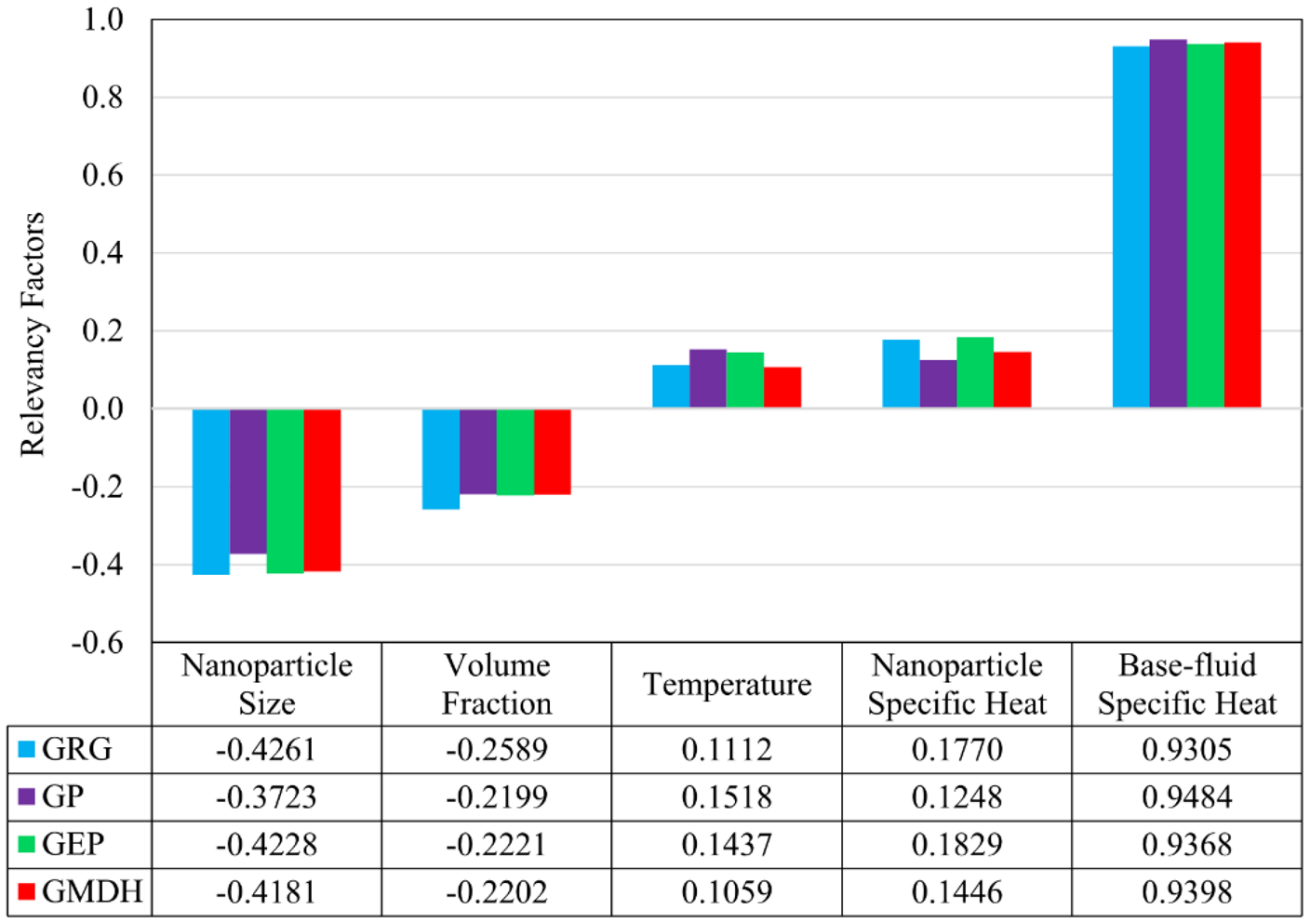
Relevancy factors between input and output variables.

One of the ways to determine the relevancy factors is to use the *Correlation* option in the *Data Analysis* tools available in the *Data* tab of *Excel* software. The relevancy factors were calculated for every one of the techniques proposed in the present research, and similar results were discovered. Figure 10 represents the relevancy factors derived for each input variable according to the estimations of all techniques. Obviously, the parameters of temperature (*T*) as well as specific heat capacities of nanoparticle (*C*_*P,np*_) and base-fluid (*C*_*P,bf*_) have a direct relationship with nanofluids’ specific heat capacity (*C*_*P,nf*_), whereas two parameters of average particle size (*d*_*np*_) and volume fraction (*ϕ*_*v*_) do not. Additionally, this figure indicates that the two parameters *C*_*P,bf*_ and *d*_*np*_ (with *r* factors of about +0.94 and −0.42, respectively) have significant impacts on estimating the target variable *C*_*P,nf*_. This issue was also discovered in the studies of Jamei et al.^84,85^, who investigated the specific heat capacity of various metal-oxide-based and carbon-based nanofluids dispersed in different base-fluids through the application of powerful machine learning techniques.

### Analyses of statistical trends

**Figure 11:**
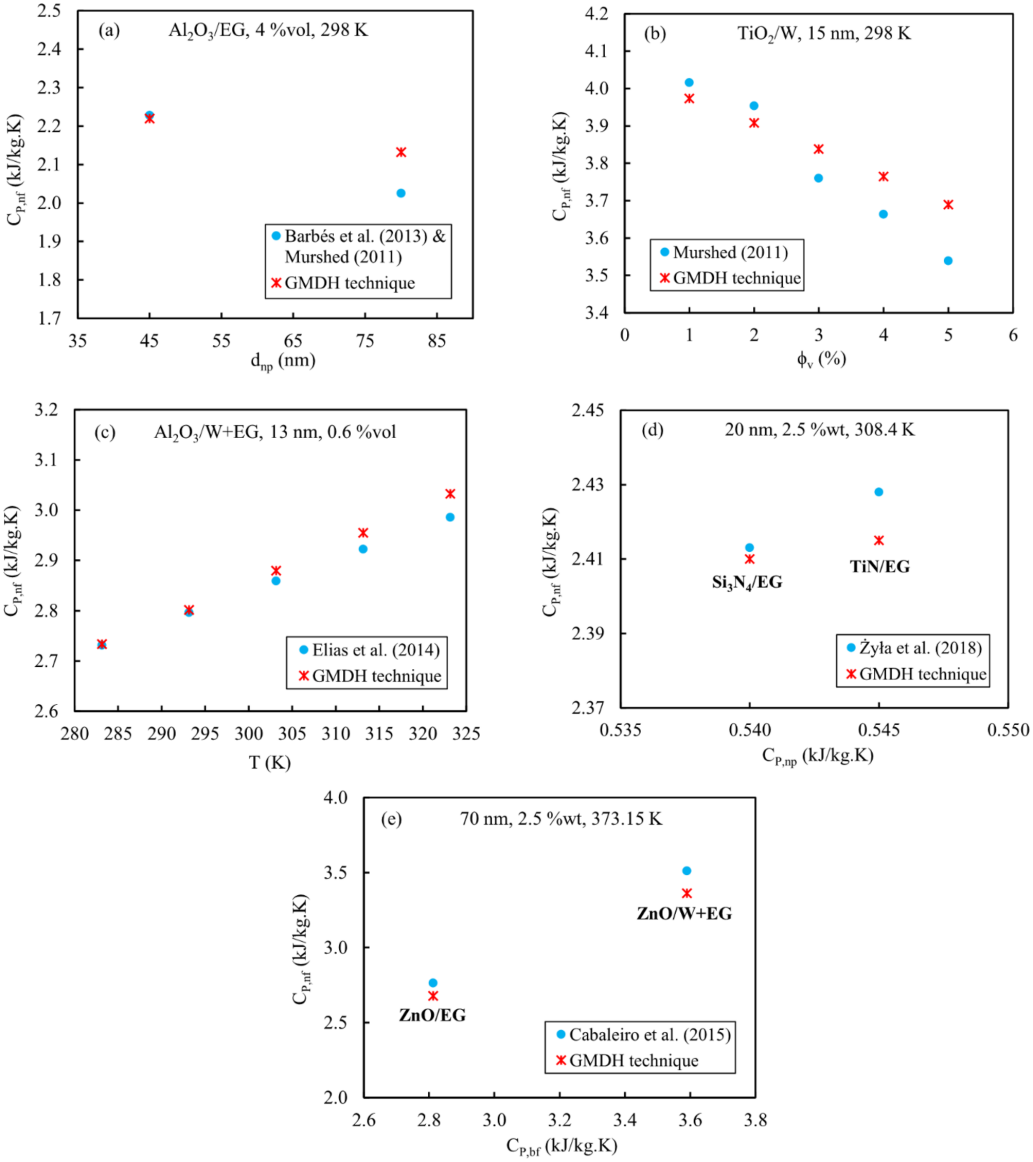
Variations of mono-nanofluids’ specific heat capacity with any input parameters.

Based on the relevancy factors outlined in the prior section, the inverse influence of average particle size (*d*_*np*_) and volume fraction (*ϕ*_*v*_) on the mono-nanofluids’ specific heat capacity (*C*_*P,nf*_) was revealed. In addition, three other variables comprising temperature (*T*), nanoparticle specific heat capacity (*C*_*P,np*_), and base-fluid specific heat capacity (*C*_*P,bf*_) had a direct proportional influence on the target variable *C*_*P,nf*_. The graphical trend analysis shown in Fig. 11 makes this issue quite evident. Here, only some experimental data and their corresponding values estimated by the best technique (i.e., GMDH) are provided.

Figure 11a depicts the decreasing trend of mono-nanofluid heat capacity (*C*_*P,nf*_) with growing nanoparticle size (*d*_*np*_). This impact was also found in the investigations of Zhang et al.^86^, Angayarkanni et al.^87^, Xiong et al.^88^, Wang et al.^89^, and Novotny et al.^90^. The underlying cause of this matter can be assigned to the fact that smaller nanoparticles may have greater surface-to-volume ratios and atomic thermal vibrational energies^86,88^. However, it was discovered that the nanoparticle size had a negligible effect, according to the study conducted by Żyła et al.^38^ on the specific heat capacity of nanofluids comprising AlN, TiN, and Si_3_N_4_ dispersed in ethylene glycol. Such a finding was also reported in Satti et al.’s^25^ research.

Based on the experimental results of Murshed^31^, Fig. 11b depicts that the values of mono-nanofluid heat capacity (*C*_*P,nf*_) reduce as the volume fraction of nanoparticles (*ϕ*_*v*_) increases. Furthermore, Satti et al.^25^ found that for small volume fractions (varying from 0.5% to 1.5%), the specific heat values of their examined nanofluids changed relatively little. In another study related to Raud et al.^91^, the specific heat values of TiO_2_/W and Al_2_O_3_/W nanofluids dropped linearly with an increase in the mass fraction of the utilized nanoparticles. The investigations of Raja et al.^92^ also disclosed that the specific heat capacity of nanofluids containing copper, silver, and aluminum nanoparticles suspended in water or ethylene glycol reduced as the nanoparticles’ concentration increased. The reason is that the solid nanoparticles have lower specific heat capacities in comparison with the base-fluids. Therefore, the more nanoparticles are dissolved in a certain amount of base-fluid, the more the specific heat capacity of the resultant nanofluid will decrease^17,93^. Similar outcomes were also attained in this field by Maghrabie et al.^94^, Moldoveanu and Minea^27^, Verma et al.^35^, and Zhou and Ni^76^.

In Fig. 11c, it is demonstrated how the specific heat value of the mono-nanofluids (*C*_*P,nf*_) varies with temperature (*T*) in such a way that there is a direct relationship between these two variables. As the temperature of the nanofluid rises, its ability to absorb and store thermal energy (without undergoing a phase transition) and as a result, the specific heat values increase. This behavior has been clearly noted in numerous studies, including Gao et al.^29^, Wole-Osho et al.^39^, Raud et al.^91^, Popa et al.^26^, Angayarkanni et al.^87^, Elias et al.^33^, Barbés et al.^21,22^, Heyhat et al.^95^, and Vajjha and Das^17,19, 20^.

Figures 11d and e illustrate the trend of mono-nanofluids’ specific heat changes against the specific heat capacities of nanoparticles and base-fluids, respectively. The direct relationship of *C*_*P,nf*_ variations with *C*_*P,np*_ and *C*_*P,bf*_ is presented for some experimental data. According to Fig. 11d, TiN-containing nanofluids have a greater specific heat capacity than those containing Si_3_N_4_ (taken from the experimental data of Żyła et al.^38^) due to the higher specific heat value of TiN nanoparticles, when considering a certain base-fluid (in this case, ethylene glycol) and keeping other parameters constant. Such an effect was also reported in the experimental study of Vijayakumar et al.^37^ regarding the difference in specific heat values of CuO/W and Al_2_O_3_/W nanofluids. As seen in Fig. 11e concerning the experimental data of Cabaleiro et al.^23^, since the specific heat value of the water-ethylene glycol mixture is greater than that of the ethylene glycol alone, the specific heat capacity of nanofluids comprising ZnO suspended in the W+EG mixture has been raised. Moreover, Akilu et al.^36^ came to a similar conclusion by evaluating the specific heat changes of SiO_2_ nanoparticles dissolved in three different base-fluids (ethylene glycol, glycerol, and ethylene glycol+glycerol). According to Fig. 11e, the base-fluid type has a considerable effect on the specific heat capacity of a nanofluid. As previously declared, the relevancy factor corresponding to the variable *C*_*p,bf*_ had the highest value (+0.94).

## Conclusions

Four soft-computing techniques were implemented on a database including 2084 data-points taken from 19 experimental studies in order to estimate the specific heat capacity of diverse mono-nanofluids (*C*_*P,nf*_) as a function of five input variables (*d*_*np*_, *φ*_*v*_, *T*, *C*_*P,np*_, and *C*_*P,bf*_). Then, four distinct high-accuracy correlations were developed, and numerous statistical and graphical error analyses were also conducted. The performance of these novel correlations was compared to the outcomes of three earlier correlations published by other researchers. The main advantage of these novel empirical correlations is that they have significantly reduced the constraints of prior correlations. As a result, they are adaptable to various oxide-based mono-nanofluids for a broad range of independent variable values.

According to the outcomes of each innovative and robust correlation developed in this research, the utilized techniques can be rated based on their accuracy as GMDH > GEP > GP > GRG. The results revealed that the correlation achieved using the GMDH technique is the most effective and appropriate one (with AAPRE = 2.4163% and R^2^ = 0.9743). However, the GEP technique has also exhibited acceptable behavior that is comparable. By applying the leverage statistical approach for the GMDH technique and distinguishing valid data, out-of-leverage data, and suspected data (outliers), the reliability and accuracy of this technique were confirmed. To recognize the relation between input and output variables available in the entire dataset, a sensitivity analysis was performed and the relevancy factors were determined for the best technique (GMDH). It was found that average nanoparticle size and base-fluid specific heat capacity are significant and influencing factors in establishing the specific heat values of mono-nanofluids. Ultimately, the variation trend of the target variable (*C*_*P,nf*_) for each of the input variables was graphically evaluated using the experimental data obtained from the previous studies and the estimated data generated by the GMDH technique. The findings of this study agree with many experimental measurements and computer-based surveys in calculating the specific heat capacity of mono-nanofluids.

### Supplementary Information

Supplementary Tables.

## Data Availability

All data have been gathered from the literature. All references used for extracting the required data have been cited in the text. However, the data will be available from the corresponding author upon reasonable request.
