# Supplementary material for: Toward empirical correlations for estimating the specific heat capacity of nanofluids utilizing GRG, GP, GEP, and GMDH
Source: Sci Rep. 2023 Nov 25;13:20763. doi: 10.1038/s41598-023-47327-x (PMC10676388; doi:10.1038/s41598-023-47327-x)
Supplement: Supplementary file 1 — Supplementary Tables. [file 41598_2023_47327_MOESM1_ESM.docx]

**Supplementary File**

Toward empirical correlations for estimating the specific heat capacity of nanofluids utilizing GRG, GP, GEP, and GMDH

**Table S1.** Detailed information on the experimental data utilized in the present work.

| **Nanoparticle Type** | **Base-fluid Type** | **Nanoparticle Fraction** | **Nanofluid Temperature** | **Nanofluid Specific Heat Capacity,**  **kJ/(kg.K)** | **Reference** |
| --- | --- | --- | --- | --- | --- |
| Al_2_O_3_ (53 nm) | W+EG | 2 - 10 %vol | 313 - 363 K | 2.348 - 3.231 | Vajjha and Das, 2008  [[1](#_ENREF_1)] |
| SiO_2_ (20 nm) &  ZnO (77 nm) | W  W+EG | 1 - 10 %vol | 315 - 363 K | 2.521 - 4.285 | Vajjha and Das, 2009  [[2](#_ENREF_2)] |
| TiO_2_ (15 nm) &  Al_2_O_3_ (80 nm) | W  EG | 1 - 5 %vol | 25 °C | 1.939 - 4.016 | Murshed, 2011  [[3](#_ENREF_3)] |
| Al_2_O_3_ (40~50 nm) | W  EG | 1 - 9.3 %vol | 296.26 - 336.44 K | 2.054 - 3.791 | Barbés et al., 2013  [[4](#_ENREF_4)] |
| CuO (23~37 nm) | W  EG | 0.4 - 3 %vol | 296.31 - 337.89 K | 2.127 - 4.094 | Barbés et al., 2014  [[5](#_ENREF_5)] |
| Al_2_O_3_ (20 nm) | W | 0.5 - 1.5 %wt | 25 - 65 °C | 3.997 - 4.249 | Teng and Hung, 2014  [[6](#_ENREF_6)] |
| Al_2_O_3_ (13 nm) | W+EG | 0.2 - 1 %vol | 10 - 50 °C | 2.568 - 3.087 | Elias et al., 2014  [[7](#_ENREF_7)] |
| MgO (35 nm) &  ZnO (40~100 nm) &  ZrO_2_ (30~60 nm) | EG  W+EG | 2.5 - 15 %wt | 243.15 - 473.15 K | 1.952 - 3.512 | Cabaleiro et al., 2015  [[8](#_ENREF_8)] |
| Al_2_O_3_ (45 nm) &  ZnO (76 nm) | W+PG | 0.5 - 6 %vol | 293 K | 2.258 - 2.574 | Satti et al., 2016  [[9](#_ENREF_9)] |
| MgO (40 nm) | W | 0.15 - 0.75 %vol | 40 - 80 °C | 4.150 - 4.189 | Kadhim et al., 2016  [[10](#_ENREF_10)] |
| CuO (42 nm) &  TiO_2_ (44 nm) &  Al_2_O_3_ (45 nm) &  SiO_2_ (10 nm) | W | 0.25 - 2 %vol | 321 K | 3.589 - 4.134 | Verma et al., 2017  [[11](#_ENREF_11)] |
| SiO_2_ (17~27 nm) | GC  EG  EG+GC | 1 - 4 %vol | 25 - 50 °C | 2.100 - 2.450 | Akilu et al., 2017  [[12](#_ENREF_12)] |
| CuO (39.1 nm)  Al_2_O_3_ (37.5 nm) | W | 0.5 - 1.5 %wt | 35 - 65 °C | 3.673 - 4.083 | Vijayakumar et al., 2017  [[13](#_ENREF_13)] |
| AlN (20 & 50 nm) &  Si_3_N_4_ (20 & 80 nm) &  TiN (20 & 50 nm) | EG | 1 - 10 %wt | 287.75 - 308.43 K | 2.172 - 2.491 | Żyła et al., 2018  [[14](#_ENREF_14)] |
| Al_2_O_3_ (29 nm) &  ZnO (70 nm) | W | 0.33 - 1.67 %vol | 25 - 65 °C | 3.502 - 4.049 | Wole-Osho et al., 2020  [[15](#_ENREF_15)] |
| MgO (40 nm) | W+EG | 0.08 - 0.2 %vol | 303 - 363 K | 2.559 - 2.898 | Choudhary et al., 2020  [[16](#_ENREF_16)] |
| ZnO (<50 nm) &  SiO_2_ (10~20 nm) | W | 0.05 - 0.2 %wt | 20 - 40 °C | 3.963 - 4.170 | Akram et al., 2021  [[17](#_ENREF_17)] |
| Al_2_O_3_ (40~50 nm) &  SiO_2_ (40~50 nm) | W | 0.1 - 0.3 %vol | 20 - 60 °C | 4.123 - 4.173 | Salameh et al., 2021  [[18](#_ENREF_18)] |
| Al_2_O_3_ (30±5 nm) | W | 0.1 %wt | 20 - 70 °C | 3.881 - 4.113 | Gao et al., 2021  [[19](#_ENREF_19)] |

**Table S2.** Descriptive statistics related to the present database.

| **Variable** | **Minimum** | **Maximum** | **Average** | **Median** | **Mode** | **Standard Deviation** | **Kurtosis** | **Skewness** |
| --- | --- | --- | --- | --- | --- | --- | --- | --- |
| *d_np_* (nm) | 10 | 80 | 45.51 | 45 | 53 | 20.088 | -1.054 | 0.172 |
| *ϕ_v_* (%vol) | 0.009 | 10 | 3.88 | 3 | 4 | 3.129 | -0.883 | 0.604 |
| *T* (K) | 253.15 | 453.15 | 328.89 | 328.29 | 293.15 | 22.485 | 4.990 | 0.802 |
| *C_P,np_* (kJ/kg.K) | 0.3914 | 1.1514 | 0.6907 | 0.7450 | 0.7650 | 0.136 | -0.523 | -0.083 |
| *C_P,bf_* (kJ/kg.K) | 2.2234 | 4.3110 | 3.5411 | 3.4091 | 4.1782 | 0.617 | -0.976 | -0.396 |
| *C_P,nf_* (kJ/kg.K) | 1.9394 | 4.2847 | 3.1904 | 3.0714 | 2.6673 | 0.636 | -1.312 | 0.124 |

**References**

[1] R. S. Vajjha and D. K. Das, "Measurements of specific heat and density of Al_2_O_3_ nanofluid," *American Institute of Physics (AIP) Conference Proceedings,* vol. 1063, no. 1, 2008, doi: <https://doi.org/10.1063/1.3027181>.

[2] R. S. Vajjha and D. K. Das, "Specific heat measurement of three nanofluids and development of new correlations," *ASME Journal of Heat and Mass Transfer,* vol. 131, no. 7, p. 071601, 2009, doi: <https://doi.org/10.1115/1.3090813>.

[3] S. M. S. Murshed, "Determination of effective specific heat of nanofluids," *Journal of Experimental Nanoscience,* vol. 6, no. 5, pp. 539-546, 2011, doi: <https://doi.org/10.1080/17458080.2010.498838>.

[4] B. Barbés, R. Páramo, E. Blanco, M. J. Pastoriza-Gallego, M. M. Piñeiro, J. L. Legido, and C. Casanova, "Thermal conductivity and specific heat capacity measurements of Al_2_O_3_ nanofluids," *Journal of Thermal Analysis and Calorimetry,* vol. 111, pp. 1615-1625, 2013, doi: <https://doi.org/10.1007/s10973-012-2534-9>.

[5] B. Barbés, R. Páramo, E. Blanco, and C. Casanova, "Thermal conductivity and specific heat capacity measurements of CuO nanofluids," *Journal of Thermal Analysis and Calorimetry,* vol. 115, pp. 1883-1891, 2014, doi: <https://doi.org/10.1007/s10973-013-3518-0>.

[6] T. P. Teng and Y. H. Hung, "Estimation and experimental study of the density and specific heat for alumina nanofluid," *Journal of Experimental Nanoscience,* vol. 9, no. 7, pp. 707-718, 2014, doi: <https://doi.org/10.1080/17458080.2012.696219>.

[7] M. M. Elias, I. M. Mahbubul, R. Saidur, M. R. Sohel, I. M. Shahrul, S. S. Khaleduzzaman, and S. Sadeghipour, "Experimental investigation on the thermo-physical properties of Al_2_O_3_ nanoparticles suspended in car radiator coolant," *International Communications in Heat and Mass Transfer,* vol. 54, pp. 48-53, 2014, doi: <https://doi.org/10.1016/j.icheatmasstransfer.2014.03.005>.

[8] D. Cabaleiro, C. Gracia-Fernández, J. L. Legido, and L. Lugo, "Specific heat of metal oxide nanofluids at high concentrations for heat transfer," *International Journal of Heat and Mass Transfer,* vol. 88, pp. 872-879, 2015, doi: <https://doi.org/10.1016/j.ijheatmasstransfer.2015.04.107>.

[9] J. R. Satti, D. K. Das, and D. Ray, "Specific heat measurements of five different propylene glycol based nanofluids and development of a new correlation," *International Journal of Heat and Mass Transfer,* vol. 94, pp. 343-353, 2016, doi: <https://doi.org/10.1016/j.ijheatmasstransfer.2015.11.065>.

[10] Z. K. Kadhim, M. S. Kassim, and A. Y. Abdul Hassan, "Effect of MgO nanofluid on heat transfer characteristics for integral finned tube heat exchanger," *International Journal of Mechanical Engineering and Technology (IJMET),* vol. 7, no. 2, pp. 11-24, 2016. [Online]. Available: <https://iaeme.com/Home/article_id/IJMET_07_02_002>.

[11] S. K. Verma, A. K. Tiwari, and D. S. Chauhan, "Experimental evaluation of flat plate solar collector using nanofluids," *Energy Conversion and Management,* vol. 134, pp. 103-115, 2017, doi: <https://doi.org/10.1016/j.enconman.2016.12.037>.

[12] S. Akilu, A. T. Baheta, K. V. Sharma, and M. A. Said, "Experimental determination of nanofluid specific heat with SiO_2_ nanoparticles in different base-fluids," in *4^th^ International Conference on the Advancement of Materials and Nanotechnology (ICAMN IV 2016)*, Langkawi, Malaysia, 2017, vol. 1877, no. 1, p. 090001, doi: <https://doi.org/10.1063/1.4999896>.

[13] M. Vijayakumar, P. Navaneethakrishnan, G. Kumaresan, and R. Kamatchi, "A study on heat transfer characteristics of inclined copper sintered wick heat pipe using surfactant free CuO and Al_2_O_3_ nanofluids," *Journal of the Taiwan Institute of Chemical Engineers,* vol. 81, pp. 190-198, 2017, doi: <https://doi.org/10.1016/j.jtice.2017.10.032>.

[14] G. Żyła, J. P. Vallejo, and L. Lugo, "Isobaric heat capacity and density of ethylene glycol-based nanofluids containing various nitride nanoparticle types: An experimental study," *Journal of Molecular Liquids,* vol. 261, pp. 530-539, 2018, doi: <https://doi.org/10.1016/j.molliq.2018.04.012>.

[15] I. Wole-Osho, E. C. Okonkwo, D. Kavaz, and S. Abbasoglu, "An experimental investigation into the effect of particle mixture ratio on specific heat capacity and dynamic viscosity of Al_2_O_3_-ZnO hybrid nanofluids," *Powder Technology,* vol. 363, pp. 699-716, 2020, doi: <https://doi.org/10.1016/j.powtec.2020.01.015>.

[16] S. Choudhary, A. Sachdeva, and P. Kumar, "Investigation of the stability of MgO nanofluid and its effect on the thermal performance of flat plate solar collector," *Renewable Energy,* vol. 147, no. 1, pp. 1801-1814, 2020, doi: <https://doi.org/10.1016/j.renene.2019.09.126>.

[17] N. Akram *et al.*, "Experimental investigations of the performance of a flat-plate solar collector using carbon and metal oxides based nanofluids," *Energy,* vol. 227, p. 120452, 2021, doi: <https://doi.org/10.1016/j.energy.2021.120452>.

[18] T. Salameh, P. P. Kumar, E. T. Sayed, M. A. Abdelkareem, H. Rezk, and A. G. Olabi, "Fuzzy modeling and particle swarm optimization of Al_2_O_3_/SiO_2_ nanofluid," *International Journal of Thermofluids,* vol. 10, p. 100084, 2021, doi: <https://doi.org/10.1016/j.ijft.2021.100084>.

[19] Y. Gao, Y. Xi, Y. Zhenzhong, A. P. Sasmito, A. S. Mujumdar, and L. Wang, "Experimental investigation of specific heat of aqueous graphene oxide Al_2_O_3_ hybrid nanofluid," *Thermal Science,* vol. 25, no. 1, pp. 515-525, 2021, doi: <https://doi.org/10.2298/TSCI190404381G>.
